# Transient Vestibulopathy in Wallenberg’s Syndrome: Pathologic Analysis

**DOI:** 10.3389/fneur.2017.00191

**Published:** 2017-05-17

**Authors:** Jorge C. Kattah, Ali S. Saber Tehrani, Sigrun Roeber, Meena Gujrati, Sarah E. Bach, David E. Newman Toker, Ari M. Blitz, Anja K. E. Horn

**Affiliations:** ^1^Department of Neurology, University of Illinois College of Medicine, Peoria, IL, USA; ^2^Center for Neuropathology and Prion Research, German Center for Vertigo and Balance Disorders, Ludwig-Maximilians-Universität, Munich, Germany; ^3^Department of Neurology, Johns Hopkins University School of Medicine, Baltimore, MD, USA; ^4^Department of Radiology, Johns Hopkins University School of Medicine, Baltimore, MD, USA; ^5^Institute of Anatomy and Cell Biology I, German Center for Vertigo and Balance Disorders, Ludwig-Maximilians-Universität, Munich, Germany

**Keywords:** lateral medullary infarction, pathology, MRI diffusion, head impulse test, transient ischemia

## Abstract

**Objective:**

To report an unusual lateral medullary stroke (LMS) associated with transient unidirectional horizontal, nystagmus, and decreased horizontal vestibulo–ocular reflex (h-VOR) gain that mimicked a peripheral vestibulopathy. MRI suggested involvement of caudal medial vestibular nucleus (MVN); however, the rapid resolution of the nystagmus and improved h-VOR gain favored transient ischemia without infarction. Decreased h-VOR gain is expected with peripheral vestibular lesions within the labyrinth or superior vestibular nerve; less frequently lateral pontine strokes involving the vestibular root entry, the vestibular fascicle, or neurons within the MVN may be responsible. The h-VOR is typically normal in LMS.

**Methods:**

Clinicopathologic examination of a 61-year-old man with an acute vestibular syndrome (AVS) and left LMS who died 3 weeks after the stroke. Postmortem brainstem analysis was performed.

**Results:**

The stroke involved the lateral medulla and pontomedullary junction, near the MVN, sparing the cerebellum and pons. To explain transient vestibular findings there are two possible hypotheses; the first would be that the MVN survived the ischemic process and would be histologically intact, and the second that vestibular afferents in the horizontal semicircular canal were ischemic and recovered after the ischemic process. Neuropathological examination showed a left LMS whose extent matched that seen by imaging. Non-ocular motor signs correlated well with structures affected by the infarction. Neurons and glia within nearby MVN were spared, as predicted by the rapid normalization of the ocular motor signs. Although unlikely, the possibility of transient intralabyrinthine arteriolar ischemia cannot be excluded. Additionally, truncal lateropulsion was due to combined lateral vestibulospinal tract and lateral reticular nucleus infarction.

**Conclusion:**

LMS may rarely be associated with an AVS that either represents or mimics a peripheral vestibulopathy. To our knowledge, this is the first neuropathologic examination of the brainstem of an LMS associated with transient vestibular findings occurring in the context of an anterior/posterior (AICA/PICA) cerebellar arterial variant stroke.

## Introduction

Following the first clinicopathologic description of a lateral medullary stroke (LMS) (1, 2), few clinicopathologic series can be found in the literature, presumably because LMS generally has a favorable outcome. HINTS examination battery in LMS is frequently indicative of central localization [normal head impulse test (HIT), presence of direction changing nystagmus, or skew deviation]. In general, persistent peripheral vestibular signs in LMS are uncommon and suggest either combined [posterior inferior cerebellar artery (PICA)/anterior inferior cerebellar artery (AICA) stroke] or a medullary/cerebellar stroke with brainstem compression (3–7). We report transient neurovestibular changes with clinical, serial imaging, and neuropathological findings in a caudal/rostral LMS patient who had sudden cardiorespiratory arrest 3 weeks after his initial stroke that presented with the acute vestibular syndrome (AVS). Initially, medial vestibular nucleus (MVN) infarction was considered; however, to explain the transient nature of the vestibular findings, we hypothesized that symptomatic, ischemic neurons in the MVN survived the ischemic process. An alternative hypothesis would be ischemia of intralabyrinthine vestibular receptor hair cells in the cupula, bipolar cells, or Scarpa ganglion neurons. This case offers unique insights into the relationship between neurovestibular signs and brainstem ischemia.

Acute vestibular syndrome is characterized by severe, continuous dizziness or vertigo, nausea, vomiting, gait instability, head motion intolerance, and nystagmus that may occur in association with peripheral or central lesions. Most LMS patients have prominent central vestibular manifestations including normal vestibulo–ocular reflex (4, 5). Delayed evolution or worsening of neurologic signs may occur, even after initial improvement of vestibular or ocular motor signs. Respiratory distress, aphonia, and need for endotracheal intubations in LMS may be a sentinel sign of respiratory failure and sudden death.

## Materials and Methods

Single LMS patient reports with postmortem neuropathologic examination, neurovestibular studies, clinicopathologic correlation, and literature review.

## Case Report

**Figure 1:**
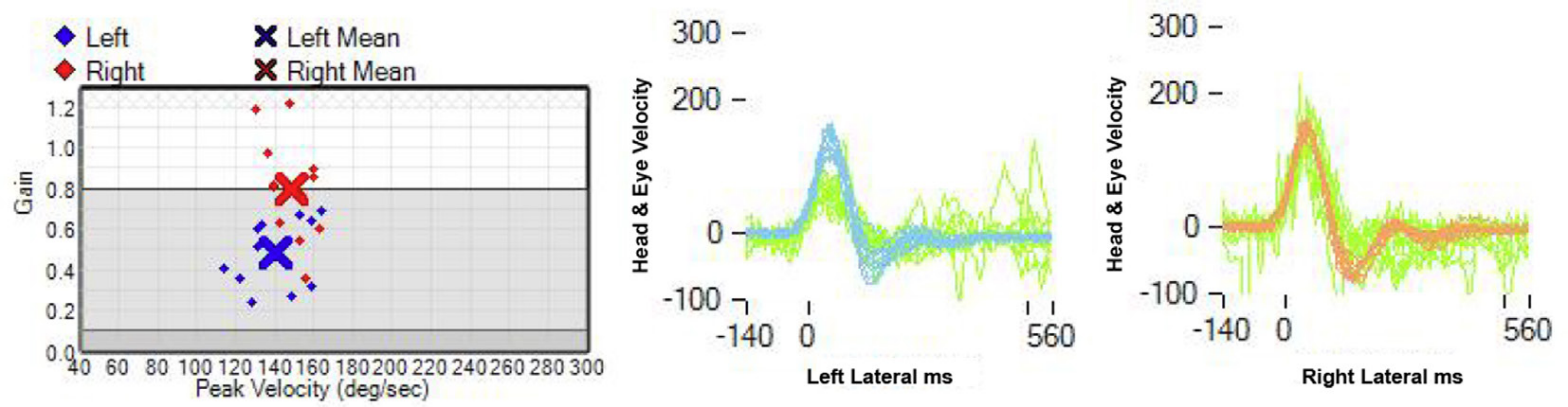
**Video-head impulse test: the gain of the left horizontal VOR is decreased: 0.48, in contrast to a right horizontal VOR gain of 0.8 (normal: 0.8)**.

**Figure 2:**
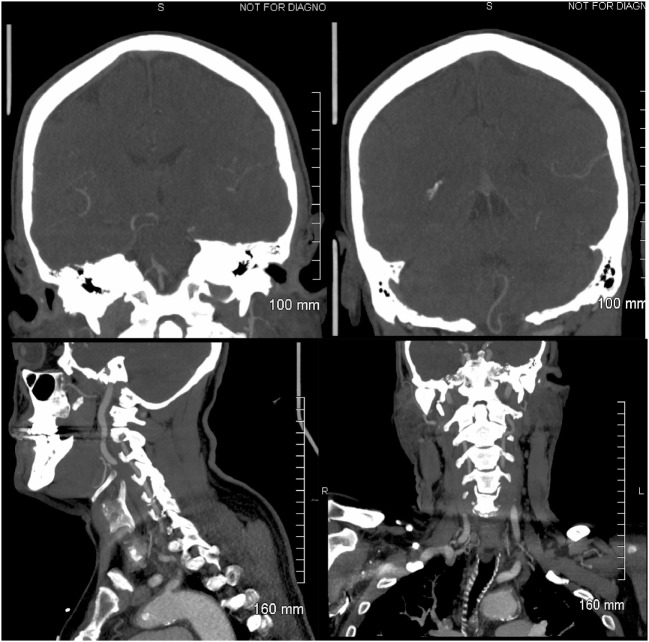
**Head and neck computerized tomography angiogram: left upper panel: coronal view of the vertebrobasilar junction**. The right superior V4 vertebral artery (VA) segment is visualized, and the origin of the right anterior inferior cerebellar artery (AICA) is observed. Retrograde flow fills the left VA. The left AICA is not visualized. Right upper panel: the left posterior inferior cerebellar artery (PICA) is imaged; it originates extra-cranially from the left V3/V4 VA and is normal, probably representing a PICA/AICA variant. Left lower panel: sagittal image of a normal left V2/V3 VA. Right lower panel: coronal view of a normal left V2/V3 VA.

Upon awakening, a 61-year-old male experienced generalized malaise. A few hours later he developed an acute AVS. He could not stand or sit without support due to intense leftward lateropulsion. Glucose intolerance was his single stroke risk factor. On examination, a left Horner’s syndrome was noted. Ophthalmoscopy and visual fields were normal. In primary gaze, we observed a conjugate horizontal right beat nystagmus that increased in right gaze, without ocular lateropulsion or skew deviation. The clinical HIT VOR was abnormal and the quantitative video-HIT (vHIT) test (ICS Impulse, GN Otometrics, Taastrup, Denmark) demonstrated decreased left gain in two consecutive trials (0.48 and 0.59, normal range >0.80), whereas right vHIT gain was normal (0.80 and 0.95). Overt refixation saccades were present (Figure 1). He did not have hearing loss to finger rubbing. Aphonia, dysphagia, hiccups, and paresis of the soft palate were noted, without tongue weakness. No limb weakness or ataxia, sensory loss, or pathologic reflexes were found. Left LMS was suspected due to presence of Horner’s, left lower cranial nerve compromise, and truncal ataxia. Additional involvement of structures in the left pontine tegmentum was considered to explain an HINTS triad consistent with peripheral lesion localization. Involvement of the rostral MVN ischemia would explain the abnormal horizontal (h)-HIT and adjacent facial nucleus, or fascicle ischemia would account for mild peripheral facial weakness. Head and neck computerized tomography angiogram (CTA) showed occlusion of the V4 segment of the left vertebral artery (VA); a robust left PICA originated extradurally from the V3/V4 junction, proximal to the occlusion (Figure 2), the right VA was hypoplastic and 50% stenotic at its origin; the right AICA artery was patent, and the left was either hypoplastic or absent. We cannot tell from the CTA if a basilar artery origin of the internal auditory artery is present; he had a left PICA/AICA variant that contributed to the circulation of the left labyrinth; the common carotid arteries were 50% stenotic bilaterally.

**Figure 3:**
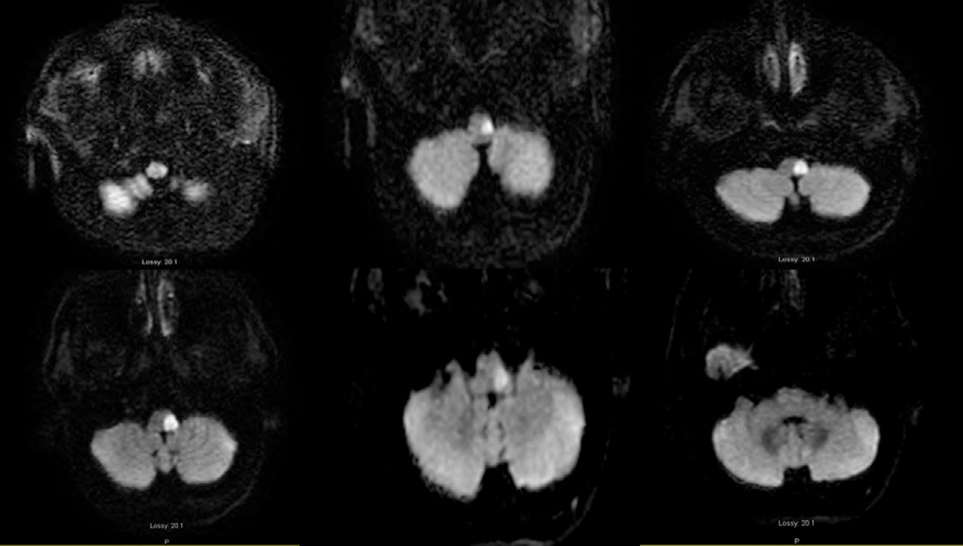
**Axial diffusion-weighted image MRI 04-25-2014: serial consecutive MRI sections of the medulla, from left: caudal to right: pontomedullary junction: restricted diffusion is present in the left lateral medulla**.

Two days later, the neurologic examination was unchanged except for resolution of the horizontal nystagmus, normalization of the h-HIT, and new impaired pin-prick sensation in the left side of the face. Progressive respiratory distress required endotracheal intubation. A brain MRI 2 days later demonstrated restricted diffusion (DWI) involving the left lateral caudal medulla with rostral extension to the pontomedullary junction, sparing the pons and the root entry of the vestibular nerve (Figure 3). An old lacunar stroke in the left putamen was noted. A cardiac work up revealed an old left bundle branch block, an ejection fraction of 38%, and septal/inferior wall hypokinesis, without evidence of acute myocardial infarction. The next morning the patient was extubated. There was no change in the neurologic examination, the h-HIT remained normal. In contrast, the left truncal lateropulsion remained severe. He remained aphonic with limited soft palate elevation. To treat dysphagia, percutaneous gastrostomy was placed.

Ten days later, worsening respiratory distress, progressive bradycardia, and occasional premature ventricular contractions developed, requiring endotracheal re-intubation. The chest X-ray showed basilar atelectasis. After treatment with antibiotics for suspected pneumonia he did well and was extubated. A repeat brain MRI showed unchanged caudal left lateral medulla DWI restriction (Figure S4 in Supplementary Material).

After recovering from the respiratory distress, he engaged in a demanding rehabilitation program. He could stand with the aid of parallel bars but had residual left lateropulsion and could not use a walker independently. His voice throughout his hospital stay was hypophonic. Unfortunately, 19 days after admission, he was found unresponsive and pulseless.

## Neuropathologic Findings

At autopsy, we found significant atherosclerotic disease including an abdominal aortic aneurysm and bilateral iliac artery stenosis. There was no acute myocardial infarction despite advanced coronary artery disease. The lungs showed no evidence of pulmonary edema, embolism, or pneumonia. A specific cause of death was not provided by the general autopsy examination. The cause of the stroke was *in situ* thrombosis of the V4 segment of the VA.

**Figure 4:**
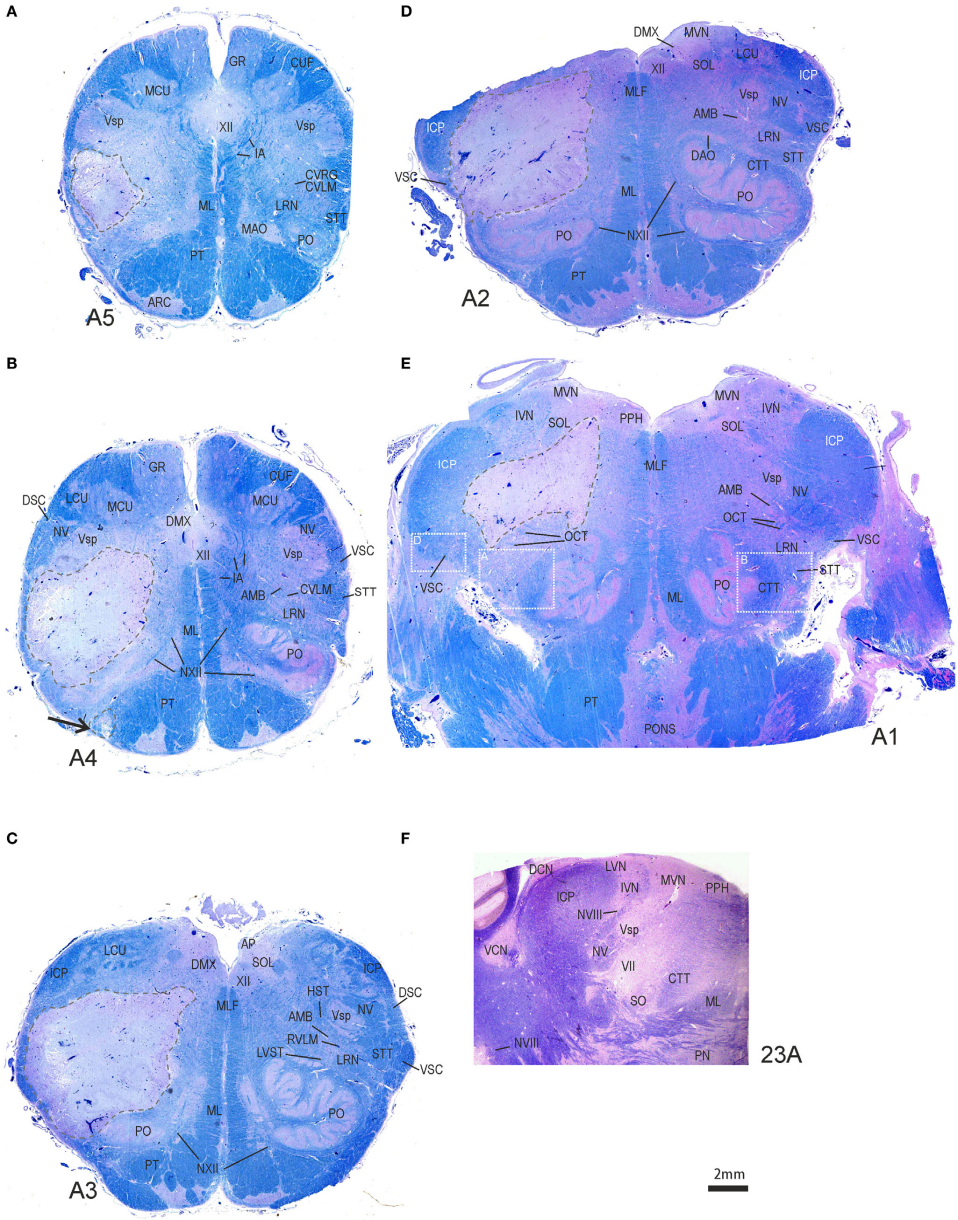
**Transversal brainstem sections from caudal to rostral stained for Luxol fast blue to demonstrate the localization and extent of the stroke**. **(A–D)** In each section, the ischemic core with total necrosis is outlined by dashed lines. Note that the left medial vestibular nucleus (MVN) and prepositus hypoglossi nucleus (PPH) are not included in the lesion **(E,F)**. Detailed views of the white boxes in **(E)** are shown in Figures S1A,B,D in Supplementary Material. AMB, nucleus ambiguous; AP, area postrema; ARC, arcuate nucleus; CTT, central tegmental tract; DCN, dorsal cochlear nucleus; DMX, dorsal motor nucleus of the vagal nerve; DSC, dorsal spinocerebellar tract; GR, gracile nucleus; HST, hypothalamic–spinal tract; IA, internal arcuate fibers; IVN, inferior vestibular nucleus; MAO, medial accessory inferior olive; LCU, lateral cuneate nucleus; MCU, medial cuneate nucleus; ML, medial lemniscus; MLF, medial longitudinal fascicle; NV, trigeminal nerve; NVIII, vestibular nerve; NXII, hypoglossal nerve; PN, pontine nuclei; PO, principal olive; PT, pyramidal tract; RVLM, rostral ventrolateral medulla; SOL, solitary nucleus; STT, spinothalamic tract; VCN, central cochlear nucleus; VSC, ventral spinocerebellar tract; Vsp, spinal trigeminal nucleus; VII, facial nucleus; XII, hypoglossal nucleus.

The brain showed significant atherosclerosis affecting the major cerebral arteries. The left lateral medulla showed marked softening of the tissue with gray–yellow discoloration. Macroscopically, the lesion did not cross the midline and was dorsal to the inferior olivary nucleus; the left VA showed advanced narrowing of the lumen, and the right was hypoplastic with focal narrowing. The basilar artery showed mild atherosclerotic changes. The right AICA was patent; the left was not described. Other than a remote lacunar infarct in the left putamen, the findings relevant to the clinical presentation were localized to the left lateral medulla. The brainstem was sectioned into 5-mm slices and embedded in paraffin following formalin fixation. Brainstem sections were stained with hematoxylin and eosin, Luxol fast blue (LFB), and immunostained for non-phosphorylated neurofilaments, glial fibrillary acid protein, and CR3/43, a marker for activated microglia. The stroke involved the left lateral medulla and affected the structures shown in tissue sections in Figure 4 and Figures S1–S3 in Supplementary Material, which are also listed in Table S1 in Supplementary Material. The stroke core involved the lateral reticular nucleus (LRN), the principal inferior olive, the spinal trigeminal nucleus, internal arcuate fibers, spinothalamic tract (STT), a small component of the lateral pyramidal tract, and the lateral vestibulospinal tract (LVST) as it traversed dorsal to the inferior olive (Figure 4). Microscopic examination of the stroke core showed necrosis with dense infiltration by foamy macrophages. The adjacent parenchymal tissue showed edematous vacuolization, numerous eosinophilic axonal swelling, and reactive astrocytes. Histologically, the neurons and neuropil in the left MVN appeared intact (Figures S1C,D in Supplementary Material), without any substantial microglia activation or gliosis in comparison to the other side (Figures S2E,F in Supplementary Material). A high number of axonal spheroids involved the olivocerebellar fibers that traverse the LRN and those of the STT (Figures S3A,B in Supplementary Material).

Immunostaining of perineuronal nets, a condensed form of the extracellular matrix, did not identify signs of ischemia (8). This observation does not rule out transient ischemia because affected perineuronal nets may have recovered within a 3-week interval (9). The vestibular nerve that is visible in the brainstem sections at its course to the vestibular nuclei showed no obvious signs of nerve ischemia or spheroids. Testing for Wallerian degeneration of the vestibular nerve was not possible because the tissue was not fixed in glutaraldehyde and not embedded in epoxy resin.

## Background

The first pathologic examination of LMS was described by Wallenberg (1, 2), and large reviews of the findings have been recently published (10). To the best of our knowledge, the neuropathology of the syndrome has been described only once in a retrospective clinicopathologic correlation series (11). A recent report listed cases from the Japanese literature (12). Although the LMS prognosis may not be as benign as is generally assumed (13), most LMS patients recover in the short term (14). In a large LMS series, however, 1- and 5-year survival rates were reported as 84 and 54%, respectively (15). The risk of sudden death in LMS due to cardiorespiratory arrest has been previously noted, presumably related to compromise of medullary respiratory and vasomotor centers (13–15) or co-existent coronary disease (16).

The main focus of our pathologic analysis was to provide a clinicopathologic investigation of the extent of the LMS core and to study the anatomic structures responsible for the transient abnormal vestibulo–ocular and the persistent vestibulospinal findings in this patient. One aim in this study was to determine if rapid recovery of the abnormal ocular findings was related to preservation of neurons within the MVN and peripheral vestibular structures or both. To our knowledge, the neuronal morphology of transient ischemia of vestibular structures in LMS has not been previously studied in humans.

## Discussion

The presenting symptoms and findings in our patient included an AVS associated with severe left axial lateropulsion, aphonia, and left Horner’s syndrome, clearly pointing to brainstem localization. The neuro-otologic examination unexpectedly showed a positive h-HIT and unidirectional, contralateral, horizontal nystagmus, typical of peripheral vestibular involvement. Furthermore, two consecutive vHIT recordings unexpectedly showed decreased VOR gain, thus localizing to either a peripheral vestibular labyrinth or an ischemic MVN mimicking a peripheral vestibulopathy (Figure 1) (6, 17).

The possibility of labyrinthine ischemia to explain the abnormal HIT VOR responses was entertained in our patient; however, the CTA demonstrated a patent right AICA that originated from the basilar artery and a robust left PICA/AICA probably a contributor to the vascular supply of the left labyrinth (Figure 2). The great majority of AVS due to labyrinthine ischemia have occurred with AICA strokes (18–20). One series of non-AICA strokes and unilateral deafness identified cochlear infarction in 7 out of 685 patients with brainstem strokes, 5 of them with PICA territory infarction, and 2 of them with severe sensorineural hearing loss (21, 22). Isolated vertigo, presumably of peripheral origin was found in 4 of 82 patients with AICA strokes by the same research group (6, 21). Combined cochleovestibular loss is the most frequent finding in labyrinthine infarction because the apex of the cochlea is more sensitive to ischemia than the vestibular labyrinth (21). Moreover, hearing loss or deafness and imaging/neuropathologic evidence of AICA territory infarction were not present in our patient but were noted in three previous reported AICA occlusions with labyrinthine infarction; two with cochlear–vestibular loss (18, 20) and one with isolated anterior vestibular artery ischemia and acute lateral pontine stroke (19). DWI signal changes involving the left vestibular nerve were not found in our patient with serial MRI studies (20). The internal auditory artery had a PICA origin in 3 out of 100 temporal lobe dissections (23) or a direct origin from the basilar artery (24). The limitations of the CTA in this case do not allow a better analysis of the vasculature; the autopsy specimen did not include a description of the left AICA, which may be explained by the PICA/AICA abnormality.

To explain the presenting nystagmus characteristics (unidirectional, horizontal, and worse with gaze toward the fast phase) and impaired horizontal vestibulo–ocular reflex (h-VOR) gain, infarction of the MVN at the pontomedullary junction should be considered. MVN involvement in LMS has been previously reported in neuropathologic LMS studies (11, 12, 25) and can be associated with abnormal h-HIT VOR and peripheral-type nystagmus (26). Experimental global ischemia in a murine animal model has shown significant vulnerability of the MVN to ischemia (27). The initial MRI in our patient (Figure 2) showed rostral extension of the infarct and provided direct imaging evidence for the proposed lesion localization (26). However, the nystagmus gradually resolved in the ensuing 48 h and the vHIT normalized clinically, suggesting that MVN neurons probably survived the ischemic process, despite the fact that the vestibular signs persisted well beyond 24 h. This was later confirmed pathologically.

An alternative explanation can be an association of the LMS with an ipsilesional partial labyrinthine infarction or ischemia sparing the brainstem parenchyma. The labyrinth is normally supplied by AICA but in our patient it was supplied by an AICA/PICA variant. In such case, the peripheral labyrinth, which is the only relevant location to account for the neuro-otologic findings that was not examined pathologically in our case, could have been a target of transient ischemia. Additional vestibular tests that could contribute to lesion localization could not be performed in an intubated patient (6). Generally, the cochlea is very sensitive to ischemia and hearing loss/deafness is frequently found but was not noted in our patient. Previous temporal bone examination performed in labyrinthine infarctions centered in the semicircular canal cupula and utricular macula, sparing the cochlea has not being described to our knowledge (18, 21).

A recent series of 172 LMS patients identified 18 isolated vestibular syndromes and provided a clinico-radiologic correlation (5). Just five of the reported patients had strokes with DWI signal changes compatible with involvement of the MVN. These patients displayed horizontal nystagmus, a positive h-HIT, caloric weakness, and gaze direction changing nystagmus. In this series, vHIT tests showed bilaterally decreased VOR gain, worse on the side of the lesion. Skew deviation, ocular tilt reaction, and other abnormalities of otolith–ocular function were also present. The h-horizontal VOR is generally normal in LMS (3–5). Accordingly, persistent peripheral vestibular signs in LMS suggest rostral extension of the stroke. A combined (PICA/AICA stroke) and less commonly combined medullary/cerebellar stroke with brainstem compression need to be evaluated. We excluded these considerations in our case by the rapid resolution of the nystagmus, normalization of the h-VOR, and lack of additional DWI signal changes in a second MRI (Figure S4 in Supplementary Material).

The severe truncal lateropulsion in our case was attributed to combined LVST and LRN infarction. The LRN is a key nucleus in the coordination of posture; it receives otolith vestibular (28), proprioceptive spinal input, projections from sensorimotor cortex, superior colliculus, and red nucleus (29–32). Efferent projections are primarily to cerebellar cortex with collaterals to deep cerebellar nuclei to regulate postural reflexes, thus, the LRN is important in the maintenance of limb and neck muscle tone (28–30, 33). In spinocerebellar ataxia type 3 severe destruction of LRN is common, affecting truncal balance (34). Experimental LRN lesions in cats cause severe postural deficits (35). Estimation of truncal lateropulsion severity and duration due to an isolated LRN, inferior cerebellar peduncle, or descending LVST versus different combinations of these structures is impossible from the current available literature. Our patient with an initially abnormal DWI restriction affecting the rostral medulla eventually had histologically normal rostral MVN and prepositus hypoglossi neurons, glial cells, and nerve fibers, correlating with a clinically milder vestibular presentation: transient unidirectional nystagmus, unilaterally abnormal vHIT, followed by a rapidly improving VOR gain, all compatible with transient ischemia. Pathologically, a stroke consists of an infarct core and surrounding ischemic tissue (ischemic penumbra) (36, 37). Ultimately, the extent of morphologic injury determines functional outcome. The brief duration of the vestibular findings suggests that the severity of the ischemia was mild and correlates with lack of neuropathologic tissue signs of ischemia in all vestibular structures examined. Normalization of the HIT and resolution of the nystagmus suggests also that the peripheral labyrinth function was restored.

The main limitations of this study relate to the lack of temporal lobe histopathology. In addition, a detailed pathologic analysis of the brainstem vasculature is not available, because of the patient’s unanticipated outcome; the autopsy was performed without clinician’s participation. Notwithstanding these limitations, the data are in favor of MVN transient ischemia, which will need to be confirmed in future cases. In our opinion, a neuropathologic evaluation of posterior fossa strokes should always include temporal bone examination.

## Conclusion

Although there are very few reports of AICA/PICA strokes (7), the clinical analysis of stroke pathogenesis include the location of the stroke core, and the surrounding penumbra and peripheral ischemia, clinicians applying these concepts to an LMS stroke associated with transient vestibular signs, regardless of the location of the vestibular pathway lesion face the following outcomes: delayed DWI signal (symptomatic ischemia without infarction), prolonged, albeit reversible ischemia in the periphery of the stroke and persistent, irreversible infarction with persistent positive DWI signal, and neuropathologic confirmation. Importantly, recent definitions of “transient ischemia” suggest it usually lasts minutes, not hours, and certainly not days (38, 39). When combined with evidence of delayed false-negative DWI imaging in patients with brainstem ischemia nearing 48 h suggests a higher tolerance threshold to ischemia (40). Rapidly reversible DWI signal changes in a stuttering stroke were documented and followed subsequently by stroke (41). Finally, whereas resolution of the nystagmus and abnormal vHIT in our patient coincided or correlated with improved DWI signal intensity in the region of the MVN, persistent DWI signal in the lateral medulla correlated with otherwise unchanged neurologic abnormalities (Figure S4 in Supplementary Material).

In summary, we describe a clinicopathologic correlation of a patient with a left LMS associated with signs of an acute peripheral vestibulopathy. The severe truncal lateropulsion was attributed to combined LVST and LRN infarction. The transient vestibular findings may either represent reversible intralabyrinthine ischemia or reversible MVN ischemia that persisted for 48 h without infarction. In previous LMS or PICA stroke series the occurrence of a labyrinth vascular syndrome is distinctly uncommon and has not been pathologically studied (21). Unless there is major breakthrough in the imaging of the labyrinth, our experience highlights the urgent need for temporal bone histopathologic examination in autopsy studies of AICA, PICA, and basilar artery strokes. Regardless of the location of the vestibular lesion in our case, the rapid resolution of the nystagmus and normalization of the HIT may correlate with recent data supporting increased tolerance to ischemia in brainstem syndromes (38, 39) and perhaps the possibility of thrombolysis beyond the 3- to 4.5-h window.

## Ethics Statement

This is a case report. Consent was obtained for reporting the findings. Written informed consent was obtained from the patient.

## Author Contributions

JK contributed to study concept and design, prepared the initial manuscript version; acquisition of data; analysis and interpretation; critical revision of the manuscript for intellectual content; and study supervision—agreed to be accountable for all aspects of the work. AT contributed to acquisition of data; analysis and interpretation; and critical revision of the manuscript for intellectual content—agreed to be accountable for all aspects of the work. SR contributed to acquisition of data, neuropathologic examination, and special tissue staining; analysis and interpretation of clinical, pathologic, and clinicopathologic correlation; and critical revision of the manuscript for intellectual content—agreed to be accountable for all aspects of the work. MG performed the initial autopsy and the first neuropathologic examination; contributed to analysis and interpretation; and approved the final manuscript—agreed to be accountable for all aspects of the work. SB reviewed the initial autopsy and the first neuropathologic examination content; contributed to analysis and interpretation; and approved the final manuscript—agreed to be accountable for all aspects of the work. DT contributed to acquisition of data; analysis and interpretation; critical revision of the manuscript for intellectual content; and approved the final version of the manuscript—agreed to be accountable for all aspects of the work. AB contributed to acquisition of radiologic and imaging data; analysis and interpretation; and approved the final manuscript—agreed to be accountable for all aspects of the work. AH contributed to acquisition of data, neuropathologic examination, and special tissue staining; labeled all the anatomic structures; analysis and interpretation of anatomical, pathologic, clinical, and clinicopathologic correlation; critical revision of the manuscript for intellectual content; and study supervision—agreed to be accountable for all aspects of the work.

## Conflict of Interest Statement

The authors declare that the research was conducted in the absence of any commercial or financial relationships that could be construed as a potential conflict of interest.

## Supplementary Material

Figure S1 **Transversal section of the brainstem through the caudal pons stained for Luxol fast blue to demonstrate the location of the magnocellular medial vestibular nucleus (MVNm) containing the secondary vestibulo–ocular neurons (A)**. The detailed view of a neighboring section immunostained for non-phosphorylated neurofilaments (NP-NF) shows numerous axonal spheroids within the inferior cerebellar peduncle (ICP) **(B)**. The high-power magnifications of HE-stained sections did not reveal any signs of neuronal degeneration within the parvocellular (MVNp) **(C)** and magnocellular MVNm **(D)**. Scale bar = 200 µm in **(B)**, scale bar = 50 µm in **(D)** [applies to **(C,D)**]. For abbreviations, see legend of Figure 3; LVN, lateral vestibular nucleus; MCP, medial cerebellar peduncle; TB, trapezoid body.

Figure S2 **Detailed views of sections treated with an antibody against activated microglia (Cr3/43) show a slightly higher number of activated cells in the left (A) compared to the right inferior cerebellar peduncle (ICP) (B)**. The same antibody depicts no difference in the medial vestibular nucleus (MVN) between left **(C)** and right side **(D)**. Immunostaining for glial fibrillary acidic protein did not reveal substantial differences in highlighting reactive astrocytes between the left **(E)** and right **(F)** MVN. Scale bar = 200 µm in**(B)** [applies to **(A,B,E,F)**]; scale bar = 100 µm in **(D)** [applies to **(C,D)**].

Figure S3 **Detailed view of the rectangle fields shown in Figure 4E stained for non-phosphorylated neurofilaments (NP-NF) to demonstrate axonal spheroids as signs of axonal damage in some fiber tracts**. The photographs of corresponding areas within the ventrolateral medulla of both sides reveal a high number of NP-NF-positive axonal spheroids involving olivocerebellar fibers that traverse the lateral reticular nucleus (LRN) and those of the spinothalamic tract (STT) **(A,C)** in comparison to the unaffected side **(B)**. A high-power magnification of the axonal spheroids in the ventral cerebellar tract (VSC) is shown in **(D)**. Scale bar = 1 mm in **(A)** [applies to **(A,B)**]; scale bar = 200 µm in **(D)** [applies to **(C,D)**].

Figure S4 **Axial DWI MRI showing baseline and follow up rostral extension of the stroke (5 day interval between scans)**. The DWI signal intensity in the follow up scan is decreased compared with baseline. In contrast, the DWI signal in the remaining axial sections is unchanged (not shown).

## Abbreviations

AICA, anterior inferior cerebellar artery; AVS, acute vestibular syndrome; LRN, lateral reticular nucleus; CVRG, caudal ventral respiratory group; CVLM, caudal ventral medulla with premotor vasomotor neurons; CTA, computerized tomography angiogram; HIT, head impulse test; LMS, lateral medullary syndrome; LVN, lateral vestibular nucleus; STT, spinothalamic tract; MVN, medial vestibular nucleus; PICA, posterior inferior cerebellar artery; PO, principal olive; PPH, prepositus hypoglossi nucleus; VA, vertebral artery; vHIT, video HIT.
