# Supplementary material for: Transient Vestibulopathy in Wallenberg’s Syndrome: Pathologic Analysis
Source: Front Neurol. 2017 May 17;8:191. doi: 10.3389/fneur.2017.00191 (PMC5434105; doi:10.3389/fneur.2017.00191)
Supplement: Figure S1 — Transversal section of the brainstem through the caudal pons stained for Luxol fast blue to demonstrate the location of the magnocellular medial vestibular nucleus (MVNm) containing the secondary vestibulo–ocular neurons (A). The detailed view of a neighboring section immunostained for non-phosphorylated neurofilaments (NP-NF) shows numerous axonal spheroids within the inferior cerebellar peduncle (ICP) (B). The high-power magnifications of HE-stained sections did not reveal any signs of neuronal degeneration within the parvocellular (MVNp) (C) and magnocellular MVNm (D). Scale bar = 200 µm in (B), scale bar = 50 µm in (D) [applies to (C,D)]. For abbreviations, see legend of Figure 3; LVN, lateral vestibular nucleus; MCP, medial cerebellar peduncle; TB, trapezoid body. [file Presentation_1.PDF]

Online Supplement for "Transient Vestibular Nuclei Ischemia in Wallenberg's Syndrome. Pathologic Analysis" (Kattah, et al.)

Table S1. Structures affected in the left lateral medullary stroke.

Affected Structures	Partially Affected Structures	Spared Structures
LEFT Ambiguous Nucleus: AMB	Inferior Cerebellar Peduncle (ICP)	Arcuate Nucleus: ARC
Lateral Reticular Nucleus: LRN	Lateral portion of the pyramidal tract	Cuneate Nucleus : CUN
Caudal ventral respiratory group: CVRG	Spinocerebellar tracts	Gracile Nucleus: GR
Caudal ventrolateral medulla with premotor vasomotor neurons: CVLM	Parts of the nucleus solitarius: SOL	Medial Lemniscus: ML
Spinal trigeminal nucleus: spin V	Principal olive: PO	Medial longitudinal fasciculus: MLF
Spino-thalamic tract: STT	Internal arcuate fibers: IA	Dorsal nucleus of the vagal nerve : DMX
Hypothalamic-spinal tract: HST	Central tegmental tract: CTT	Pyramidal tract: PT
Lateral vestibulospinal tract: LVST		Prepositus hypoglossi : PPH
Medial accessory inferior olive: MOA		Medial vestibular Nucleus MVN
Dorsal accessory inferior olive: DAO		Inferior vestibular Nucleus: IVN
Rostral ventrolateral medulla: RVLM		Lateral vestibular Nucleus : LVN (not shown)
		Ventrolateral olive
		Hypoglossal nerve: XII

Abbreviation List:

AICA = anterior inferior cerebellar artery; AVS = acute vestibular syndrome; LRN = lateral reticular nucleus; CVRG = caudal ventral respiratory group; CVLM = caudal ventral medulla with premotor vasomotor neurons; CTA = computerized tomography angiogram; HIT = head impulse test; LMS = lateral medullary syndrome; LRN = lateral reticular nucleus; LVN = lateral vestibular nucleus; STT= spinothalamic tract, MVN = medial vestibular nucleus; PICA = posterior inferior cerebellar artery; PO = principal olive; PPH = prepositus hypoglossi nucleus, VA = vertebral artery; vHIT = video HIT

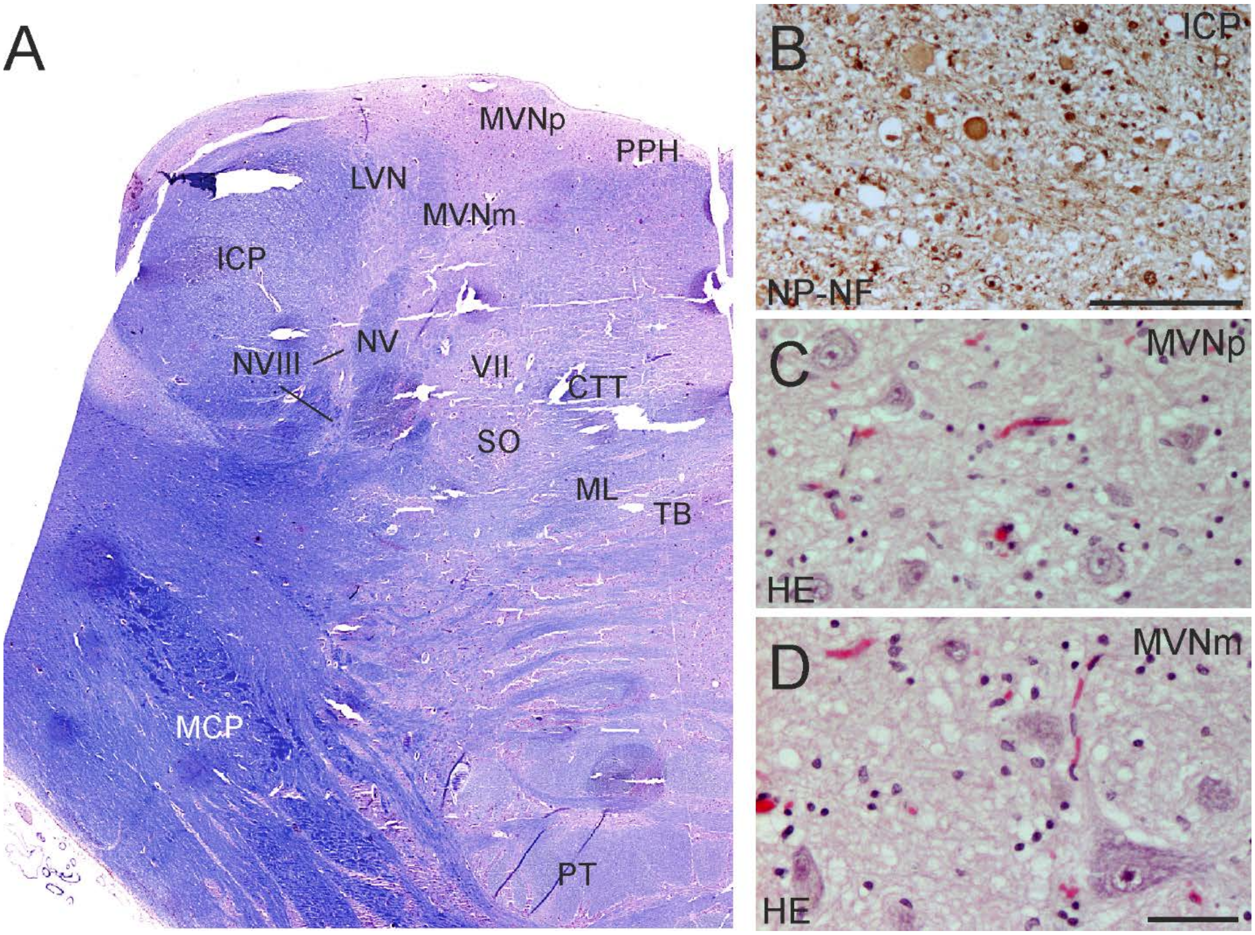

Figure S1

Transversal section of the brainstem through the caudal pons stained for LFB to demonstrate the location of the magnocellular medial vestibular nucleus (MVNm) containing the secondary vestibulo-ocular neurons (A). The detailed view of a neighboring section immunostained for non-phosphorylated neurofilaments (NP-NF) shows numerous axonal spheroids within the inferior cerebellar peduncle (ICP) (B). The high-power magnifications of HE-stained sections did not reveal any signs of neuronal degeneration within the parvocellular (MVNp) (C) and magnocellular MVNm (D). Scale bar = 200µm in (B), scale bar = 50µm in D (applies to C, D).

Abbreviations see legend in Figure 4: LVN = lateral vestibular nucleus; MCP = medial cerebellar peduncle; TB = trapezoid body

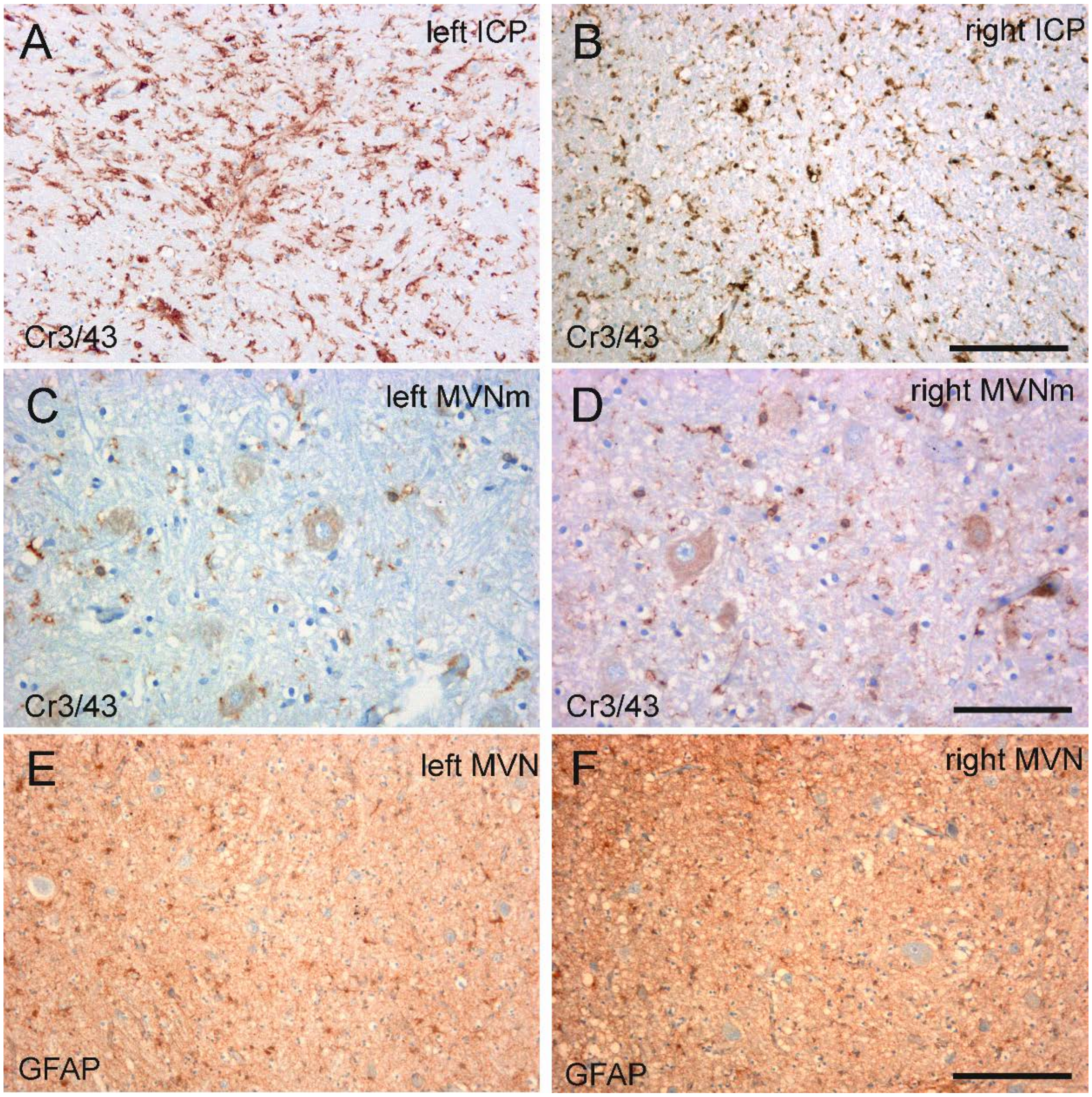

Figure S2

Detailed views of sections treated with an antibody against activated microglia (Cr3/43) show a slightly higher number of activated cells in the left (A) compared to the right inferior cerebellar peduncle (ICP) (B). The same antibody depicts no difference in the medial vestibular nucleus (MVN) between left (C) and right side (D). Immunostaining for glial fibrillary acidic protein (GFAP) did not reveal substantial differences in highlighting reactive astrocytes between the left (E) and right (F) MVN. Scale bar = 200µm in B (applies to A, B, E, F); scale bar = 100µm in D (applies to C, D).

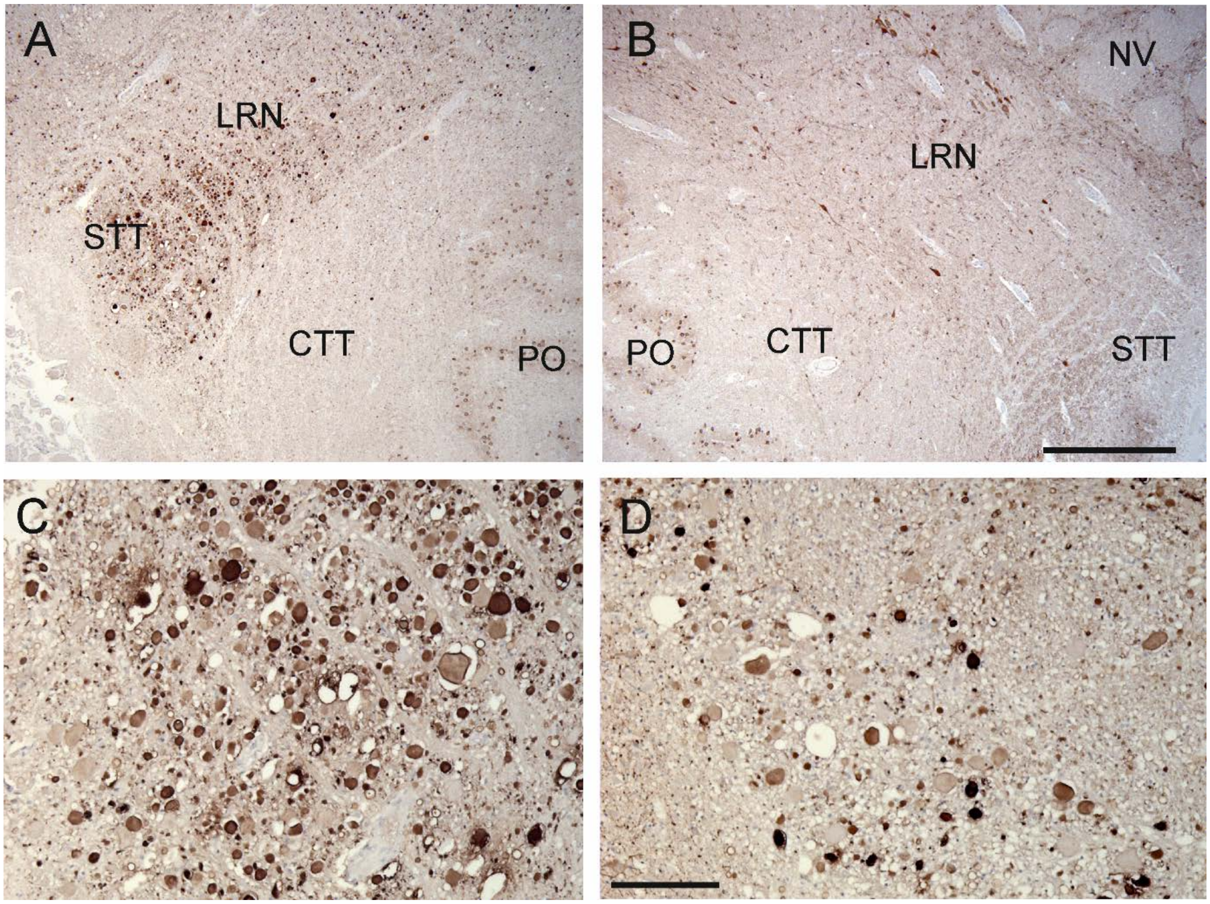

Figure S3

Detailed view of the rectangle fields shown in Figure IIE stained for non-phosphorylated neurofilaments (NP-NF) to demonstrate axonal spheroids as signs of axonal damage in some fiber tracts. The photographs of corresponding areas within the ventrolateral medulla of both sides reveal a high number of NP-NF-positive axonal spheroids involving olivo-cerebellar fibers that traverse the lateral reticular nucleus (LRN) and those of the spinothalamic tract (STT) (A, C) in comparison to the unaffected side (B). A high-power magnification of the axonal spheroids in the ventral cerebellar tract (VSC) is shown in (D). Scale bar = 1mm in A (applies to A, B); scale bar in D = 200µm (applies to C, D).

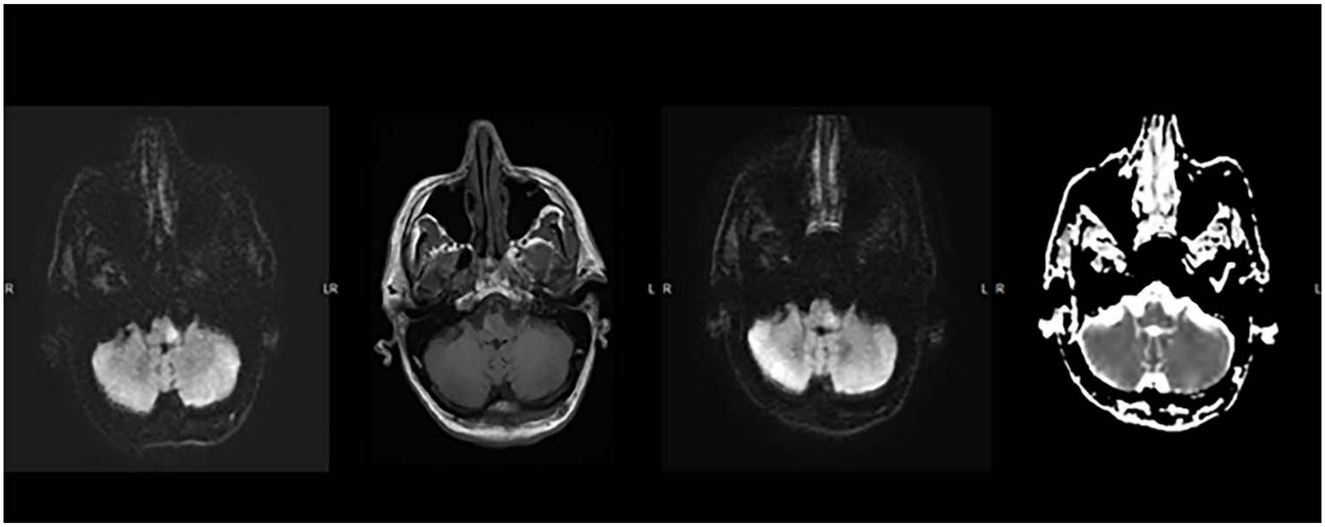

Figure S4

Axial DWI MRI showing baseline and follow up rostral extension of the stroke (5 day interval between scans). The DWI signal intensity in the follow up scan is decreased compared with baseline. In contrast, the DWI signal in the remaining axial sections is unchanged (not shown).
